# Oncological Outcomes and Genomic Features of Gastric-Type Endocervical Adenocarcinoma, the Most Aggressive and Common HPV-Independent Cervical Cancer

**DOI:** 10.3390/cancers18020320

**Published:** 2026-01-20

**Authors:** Ming Du, Zhen Zheng, Peiyao Lu, Weidi Wang, Dongyan Cao, Jiaxin Yang, Ming Wu, Lingya Pan, Xiaowei Xue, Wenze Wang, Fang Jiang, Yang Xiang

**Affiliations:** 1 Department of Obstetrics and Gynecology, Peking Union Medical College Hospital, Chinese Academy of Medical Sciences and Peking Union Medical College, Beijing 100730, China; b2023001104@pumc.edu.cn (M.D.); b2024001116@pumc.edu.cn (Z.Z.); lupy18@student.pumc.edu.cn (P.L.); b2024001267@pumc.edu.cn (W.W.); caodongyan@pumch.cn (D.C.); yangjiaxin@pumch.cn (J.Y.); wuming@pumch.cn (M.W.); panly@pumch.cn (L.P.); jiangfang@pumch.cn (F.J.); 2 Department of Pathology, Peking Union Medical College Hospital, Chinese Academy of Medical Sciences and Peking Union Medical College, Beijing 100730, China; xuexiaowei@pumch.cn (X.X.); wangwz@pumch.cn (W.W.); 3 Department of Obstetrics and Gynecology, National Clinical Research Center for Women’s Health and Obstetric & Gynecologic Diseases, Beijing 100730, China

**Keywords:** HPV-independent cervical cancer, gastric-type endocervical adenocarcinoma, genomic alterations, prognosis

## Abstract

**Background/Objectives**: In order to develop a comprehensive understanding of gastric-type endocervical adenocarcinoma (GEA), an increasingly prevalent HPV-independent cervical cancer, we summarized clinicopathological information and performed prognostic analysis. **Methods**: A total of 182 patients diagnosed with GEA at our center during the period 2014–2025 were included in this study. Nineteen GEA cases, 6 HPV-independent non-GEA cases, 59 HPV-associated usual endocervical adenocarcinoma cases, and 66 squamous cell carcinoma cases from online database were also included. **Results**: Vaginal bleeding (39.56%) and watery discharge (35.16%) were the most common symptoms. As many as 21.43% of patients had no specific complaints, and 80% of GEA showed no distinct mass through gynecological examination. A total of 64% of GEA were stage IIB–IV at diagnosis, with a 5-year survival of 41% versus 85% for stage I–IIA (*p* < 0.05). The rate of lymphovascular space invasion (LVSI), lymph node metastasis, and ovarian metastasis were 49.64%, 42.00%, and 29.29%, respectively. The 5-year survival and recurrence rates after primary therapy were 57% and 23%, respectively. For GEA treatment, surgery might be associated with improved overall survival for the population at stage III–IV. Survival analysis identified deep infiltration depth (≥2/3), a maximum diameter of the tumor (MDOT) of ≥3 cm, and ovary metastasis as potential indicators of worse OS and PFS for whole patients. Additionally, ovary metastasis indicated poor PFS and OS for stage I–II. Genomic information TP53 mutation, PTEN deletion and STK11 mutation might be the most prevalent genomic alterations. **Conclusions**: These findings indicated GEA as an aggressive cervical cancer, with high rate of lymph node metastasis, high recurrence rate and short 5-year survival. Ovary metastasis reflected advanced disease burden and surgery might be associated with improved survival in advanced stage. For genomic information, GEA showed genetic heterogeneity and a low level of genomic instability.

## Simple Summary

As the most common HPV-independent cervical cancer, gastric-type endocervical adenocarcinoma is more aggressive than HPV-associated squamous or adenocarcinoma and thus may constitute the last phase in the WHO’s effort to eliminate the disease. However, its genomic and clinicopathological information are limited compared with that for HPV-associated cervical cancer. This study contributes valuable data on treatment regimen, oncological outcomes, prognosis analysis, and genomic alterations. The prognostic value of ovary metastasis provided in this study affords clinicians detailed information on GEA with which to better diagnose and treat this disease. Moreover, the genomic information provided herein serves as evidence for the benefits of targeted therapy, which will be valuable for future practice and research in the field of GEA.

## 1. Introduction

According to the 2024 global cancer statistics, cervical cancer has become the third leading cause of cancer mortality in females since 2019 [1]. The stagnant death rate has been attributed to the emergence of HPV-independent (HPVI) adenocarcinoma, despite HPV vaccine exposure and screening programs [2,3,4]. Gastric-type endocervical adenocarcinoma (GEA) is the most common subpopulation among HPVI types according to the World Health Organization (WHO) 2020 nomenclature and the International Endocervical Adenocarcinoma Criteria and Classification (IECC) 2018 [5,6]. The survival rate for GEA was significantly lower than that of squamous cell carcinoma (SCC), HPV-associated (HPVA) adenocarcinomas, and other HPVI adenocarcinomas [2,7,8,9]. Thus, overcoming GEA may be the last phase in the war against cervical cancer.

However, multiple dilemmas remain to be overcome in GEA management, owing to its statistical rarity. First, preoperative diagnosis accuracy of GEA is often impaired by physicians failing to distinguish between precursor and malignant lesions [10,11], between cervical and non-cervical diseases (such as ovarian cancer) [12,13,14], and between pure GEA and GEA with squamous differentiation or mixed squamous elements [15,16,17]. Second, due to the quiet symptoms and aggressive behavior, more than 50% of GEA cases were diagnosed at the late stage following lymph node, ovary, or uterine metastasis [7,18,19]. Third, compared to the SCC and HPVA adenocarcinomas, GEA shows a significantly limited response to postoperative radiotherapy alone (RT) or concurrent chemoradiotherapy (CCRT) [3,7,20,21], neoadjuvant chemotherapy with docetaxel and carboplatin [22], and gastric cancer chemotherapy regimen [23]. Fourth, the time to recurrence (TTR) for GEA is shorter than HPVA usual-type endocervical adenocarcinoma (UEA), and relapse often occurs within 1 year after surgery [7,24]. Even in well-differentiated GEA, the locoregional recurrence rate can reach up to 23.3% [25]. Overall, finding a way to improve the early diagnosis rate and the efficacy of therapy is extremely urgent in improving GEA prognosis.

Currently, sequencing profiles and immunohistochemistry (IHC) information aid in precision diagnosis and targeted therapy. Genomic data indicates strong genomic heterogeneity in GEA, and TP53 mutation, STK11 mutation, HER2 amplification varies across different cases [26]. For targeted therapy, HER2-targeting drugs might assist in combating the HER2 amplification or overexpression in GEA based on a “basket trial” [19,27,28]. Transcriptome data indicated some crucial factors affecting GEA progress and prognosis, such as tight junctions and cell cycle-related elements [29,30]. GEA protein data concerning MUC6, HIK1083, and CLDN18 have provided inspiration for differential diagnosis [31,32,33]. Thus, considering its rarity, comparing GEA with UEA from different perspectives and uncovering the coherence and heterogeneity among GEA cases might assist us in tailoring regimens for different patients.

Herein, we collected the clinicopathological parameters, MRI features, and genomic characteristics to better understand this rare and aggressive disease. Moreover, we also analyzed the prognostic factors for PFS and OS in GEA cases.

## 2. Materials and Methods

### 2.1. Patients and Follow-Up

A total of 182 patients with a histologic diagnosis of GEA at Peking Union Medical College Hospital between 2014 and 2025 were included in this study. This study was approved by the ethics committee in Peking Union Medical College Hospital [No. K7858], and all participants independently signed the informed consent form when enrolled. The electronic records were queried for all patients, and the study design was shown in a flow chart (Figure S1). The follow-up was performed by outpatient review or telephone, and the last follow-up date for survival analyses was 7 October 2025. Overall survival (OS) was defined from the date of diagnosis to the date of death or the last follow-up. Recurrence-free survival (RFS) was defined from primary therapy to recurrence and progression-free survival (PFS) was defined as the time from the end of primary therapy to the date of the disease relapse, disease progression, or death.

Ten cases among 182 patients (5.49%) were included before 2018, and reclassification was performed according to the International Federation of Gynecology and Obstetrics (FIGO) 2018 staging system. Tumor maximum diameter, stromal invasion depth, lymph metastasis, lymphovascular space invasion and ovarian metastasis were pathologically confirmed via radical hysterectomy (*n* = 139). Regarding lymph metastasis, apart from pathological confirmation from 139 patients undergoing radical hysterectomy, 10 other patients were also considered to have lymph node metastasis based on PET/CT findings showing lymph node nodules larger than 1.5 cm with significantly elevated SUVmax value.

### 2.2. Targeted Genome Sequencing and Whole Exon Sequencing

Genomic sequencing was recommended to all GEA patients treated at our institution, and due to cost considerations, 17 patients ultimately underwent sequencing. Seven patients chose targeted genome sequencing with targeted panel according to standards and guidelines for the interpretation and reporting of sequence variants in cancer [34], using the formalin-fixed, paraffin-embedded (FFPE) tissue samples of primary tumor tissues and paired peripheral blood. Ten patients performed whole exon sequencing (WES) using fresh tissue samples of primary tumor tissues and paired peripheral blood. Targeted sequencing and WES were performed with a NovaSeq 6000 platform (Illumina, San Diego, CA, USA), using paired-end 150 bp protocols according to the manufacturer’s instructions. For targeted sequencing, the coverage of tumor tissues was no less than 1000×, and the coverage of blood samples was no less than 100×. For WES, the mean coverage of tumor tissues was 200× and the mean coverage of blood samples was 100×. All sequencing data have been uploaded to the GSA database (accession number: PRJCA049944), and the germline and somatic mutations were analyzed (details shown in Supplementary Method) and summarized in this research.

### 2.3. Online Database Analysis

A total of 19 GEA cases, 6 HPV-independent non-GEA (HPVI-non GEA) cases, 59 HPV-associated usual-type endocervical adenocarcinoma (UEA) cases, and 66 SCC cases were included in this study from the cBioPortal database (https://www.cbioportal.org/).

### 2.4. Statistical Analysis

The statistical calculations were performed using SPSS version 28.0. OS, PFS, and RFS were analyzed with Kaplan–Meier (K–M) analysis and compared with the log-rank test. Prognostic analysis for patients undergoing surgery (*n* = 139) was performed using the univariate and multivariate cox regression, and clinical variables including age, FIGO stage, MDOT, infiltrating depth, LVSI, lymph node metastasis, and ovarian metastasis were included for multivariate analysis. Treatment-selection analysis was performed using multivariate cox regression with the following approaches: For stage I–II disease (*n* = 82), the necessity of postoperative treatment was assessed by incorporating clinical variables such as age, postoperative treatment status and ovarian metastasis. For stage III–IV disease (*n* = 100), the necessity of surgery was evaluated using a model that included age and surgical status (whether surgery was performed or not). For the whole patients (*n* = 182), the necessity of surgery was evaluated using a model that included age, FIGO stage, and surgical status (whether surgery was performed or not).

## 3. Results

### 3.1. Clinicopathological Characteristics of GEA Patients

**Figure 1:**
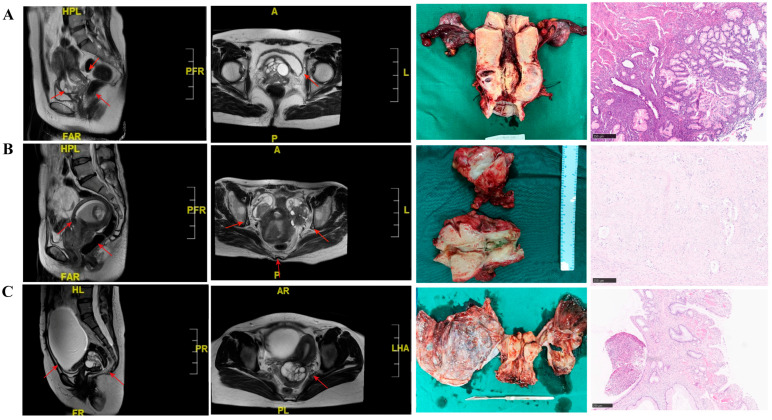
Typical MRI, gross, and histologic image features of GEA patients: (**A**) case with “cosmos” features in MRI and multi-cystic components in mass image; (**B**) case with ovary metastasis in MRI, gross, and pathologic image; (**C**) case with PJS (the patient was diagnosed with GEA but also LEGH of the endometrium, LEGH of the bilateral fallopian tubes, and mucinous cystadenoma of the ovary at the same time) (All the red arrows on the pelvic MRI images indicate the location of lesions).

A total of 182 cases were included in this study, and the detailed demographic and clinicopathological information are summarized in Table 1. The median follow-up duration was 28.5 months (range: 2.4 months–140.3 months), and 27.47% of patients (*n* = 50) were deceased at the last follow-up date. The median age at diagnosis was 49 years (range: 24–81 years), and seven patients were diagnosed with Peutz–Jeghers syndrome (PJS). The most common presentation was vaginal bleeding (39.56%) and discharge (35.16%), and 21.43% of patients were asymptomatic (Table 1). Nearly 90% of cases were HPV-negative, and approximately 60% of cases showed intraepithelial lesion or malignancy according to TCT examination (Table 1). CA199 (83/161, 51.55%) and CA125 (12/161, 7.45%) were elevated in partial cases, respectively. Representative MRI images were manifested as the cervical hypertrophic or barrel-shaped change or the “cosmos” pattern, which manifested as smaller central cystic or solid focus surrounded by larger peripheral cysts (Figure 1).

According to the FIGO 2018 staging system, 63.74% (116/182) were stage IIB–IV at diagnosis. Overall, gross examination indicated that GEA typically presented as an indurated or barrel-shaped cervix with multicystic or solid or cystic components within the tumor (Figure 1). Microscopic and imaging examination showed that lymph node metastasis was observed in 42% of patients. For patients undergoing surgery (*n* = 139), pathological examination confirmed that the malignancy lesions usually infiltrated more than two-thirds of the cervical stroma, and the tumor maximum diameter for nearly half the patients was more than 3 cm. Pathological examination also confirmed that lymph–vascular space invasion (LVSI) was pathologically observed in nearly 50% of 139 cases. Forty-one patients (nearly 30%) showed ovarian metastasis among 140 cases with available pathological confirmation, and fourteen patients had fallopian tube metastasis among these 41 cases.

Microscopically, morphology features included clear or foamy eosinophilic cytoplasm and easily identified cell membranes with varying degrees of nucleus atypia (Figure 1). Molecular features indicated that a high expression of MUC6 was detected in 91.82% of patients (*n* = 101), and mutated-p53 expression was detected in 33.85% of cases (*n* = 44).

### 3.2. Treatment Regimens of GEA Patients

The treatment regimen was tailored for 182 patients according to the stage, general condition, and affordability (Table 2). A total of 59 patients (32.41%) were stage I at diagnosis: 1 patient received CCRT alone, and 50 patients underwent radical hysterectomy alone (*n* = 12) or did so followed by CCRT or sequential chemoradiotherapy (SCRT) (*n* = 38). The neoadjuvant CCRT/SCRT/chemotherapy was performed in eight patients to shrink the tumor lesions; then, all patients finished radical hysterectomy. A total of 23 patients (12.64%) were stage II at diagnosis: 11 patients underwent radical hysterectomy alone (*n* = 11) or did so followed by CCRT or SCRT (*n* = 10); 12 patients received CCRT or SCRT alone (*n* = 8) or did so followed by surgery with postoperative therapy (*n* = 4). A total of 67 patients (36.81%) were stage III at diagnosis: 45 patients received radical hysterectomy followed by chemotherapy/SCRT/CCRT; 22 patients received chemotherapy/SCRT/CCRT alone (*n* = 18) or did so followed by surgery with postoperative therapy (*n* = 4). A total of 33 patients (18.13%) were stage IV at diagnosis: 11 patients received surgery followed by CCRT/SCRT/chemotherapy, and 22 patients underwent CCRT/SCRT/chemotherapy alone (*n* = 16) or did so followed by surgery with postoperative therapy (*n* = 6). Moreover, immunotherapy was used in 41 of the 182 patients (stage I: *n* = 6; stage II: *n* = 4; stage III: *n* = 22; stage IV: *n* = 9), and targeted therapy was used in 26 patients (stage I: *n* = 2; stage II: *n* = 1; stage III: *n* = 14; stage IV: *n* = 9).

**Figure 2:**
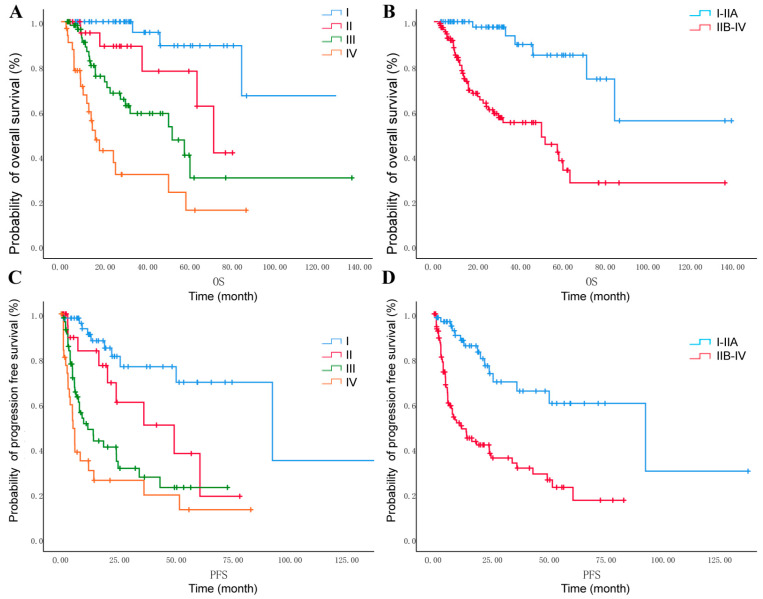
Progression-free survival (PFS) and overall survival (OS) of GEA patients: (**A**) OS for GEA according to FIGO stage (blue = FIGO stage I; red = FIGO stage II; green = FIGO stage III; orange = FIGO stage IV; Log rank *p* < 0.001). (**B**) OS for GEA with stage I–IIA versus stage IIB–IV (blue = FIGO stage I–IIA; red = FIGO stage IIB–IV; Log rank *p* < 0.001). (**C**) PFS for GEA according to stage (blue = FIGO stage I; red = FIGO stage II; green = FIGO stage III; orange = FIGO stage IV; Log rank *p* < 0.001). (**D**) PFS for GEA with stage I–IIA versus stage IIB–IV (blue = FIGO stage I–IIA; red = FIGO stage IIB–IV; Log rank *p* < 0.001).

Forty-one patients (22.53%) underwent recurrence after primary treatment, and the median TTR is 9.3 months (Table 2). For second-line regimen, 22 patients received chemotherapy combined with immunotherapy with or without targeted therapy; 4 patients underwent surgery followed by adjuvant therapy; and 1 patient received radiotherapy alone. For survival outcomes, the median OS and PFS in these 182 cases were 18 months and 8 months, respectively, and the 5-year survival rate of the 182 patients was 57%. OS and PFS were significantly different across the four stages, and the overall survival rate for stage I–IIA was significantly poorer than that of stage IIB–IV (Figure 2), with 5-year survival rates of 85% and 41%, respectively.

### 3.3. Oncological Outcomes and Survival Analysis of GEA Patients

**Figure 3:**
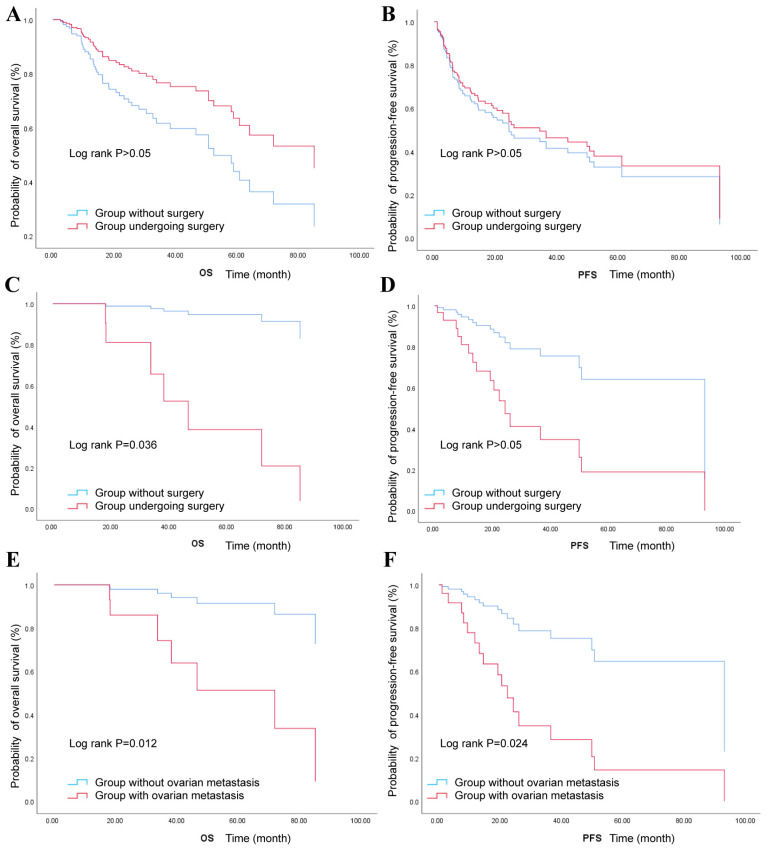
Comparison analysis of OS and PFS among GEA patients: (**A**) Comparison analysis of OS among 182 patients (red = group undergoing surgery; blue= group without surgery; Log rank *p* > 0.05). (**B**) Comparison analysis of PFS among 182 patients (red = group undergoing surgery; blue= group without surgery; Log rank *p* > 0.05). (**C**) Comparison analysis of OS among stage III–IV patients (red = group undergoing surgery; blue= group without surgery; Log rank *p* = 0.036). (**D**) Comparison analysis of PFS among stage III–IV patients (red = group undergoing surgery; blue= group without surgery; Log rank *p* > 0.05). (**E**) Comparison analysis of OS among stage I–II patients (blue = group without ovary metastasis; red = group with ovary metastasis; Log rank *p* = 0.011). (**F**) Comparison analysis of PFS among stage I–II patients (blue = group without ovary metastasis; red = group with ovary metastasis; Log rank *p* = 0.010).

For stage I–II (*n* = 82), to clarify the necessity of postoperative therapy, multivariate cox regression was performed. Comparison analysis showed that there was no significant difference in PFS, OS, and RFS between patients undergoing surgery with or without postoperative therapy at stage I–II (*p* > 0.05) (Figure S2 and Table S1). For stage III–IV (*n* = 100), to preliminarily explore whether the surgery might affect oncological outcomes, multivariate cox regression was performed among stage III and IV (Table S1). It was suggested that treatment involving surgery might be related to longer OS at stage III and IV (*p* < 0.05), with no significant difference in PFS compared with the population not undergoing surgery in these patients (Figure 3C,D). Moreover, cox analysis across all 182 cases showed that the OS and PFS of patients without surgery showed no significant difference between those of patients undergoing surgery (Figure 3A,B and Table S1).

To identify risk factors affecting prognosis for patients undergoing surgery, univariate analysis and multivariate cox regression analysis were performed in 139 cases undergoing surgery. Univariate analysis indicated that FIGO stage, MDOT (the maximum diameter of the tumor), deep infiltration depth (≥2/3), lymph node metastasis, and ovary metastasis were significant parameters for both OS and PFS, with an additional association with a worse PFS of the lymphovascular space invasion (LVSI) (Table 3). Multivariate analysis showed that FIGO stage, LVSI, infiltration depth, MDOT, and ovary metastasis were significant parameters for OS, and MDOT, infiltrating depth, and ovary metastasis were factors affecting PFS (Table 3). Considering the high frequency and additional prognostic value of ovary metastasis, multivariate analysis was performed for patients undergoing at stage I–II (Table S1). Similarly to the entire patient sample, ovary metastasis was significantly related to worse PFS and OS (Figure 3E,F).

### 3.4. Genomic Profiles of GEA Patients

**Figure 4:**
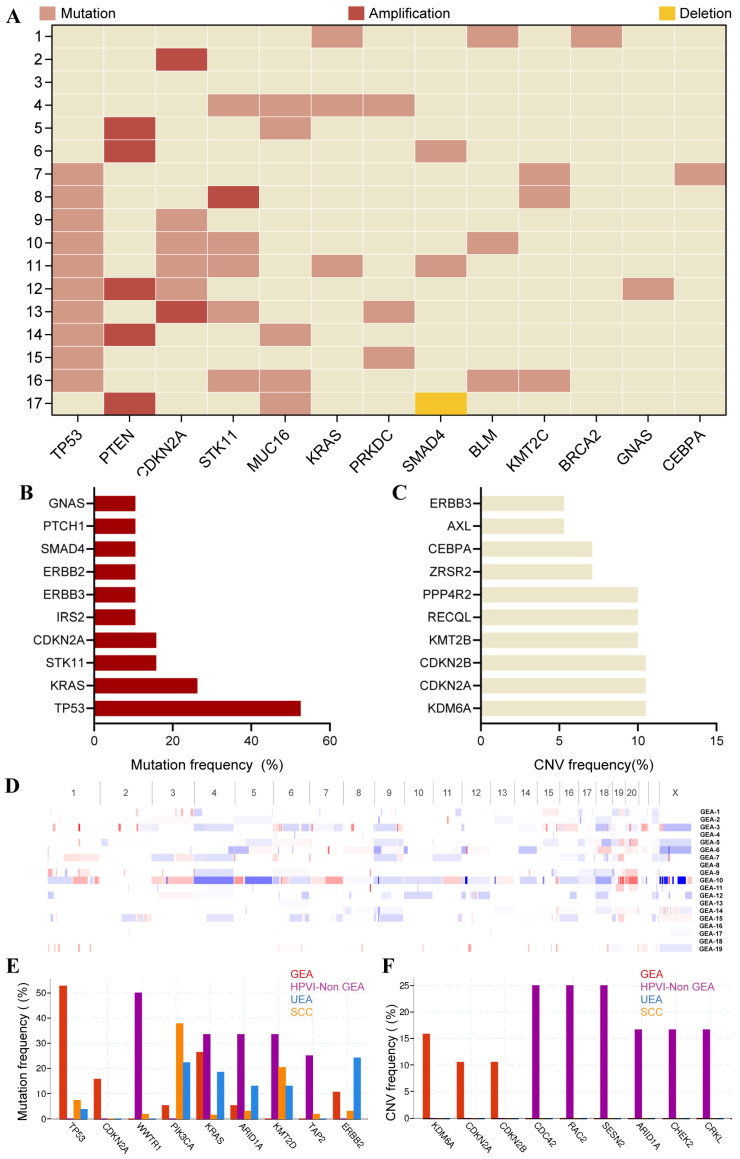
Genomic profiles of GEA patients: (**A**) Genomic alterations of 17 GEA patients from Peking Union Medical College Hospital; (**B**) The most common mutations of 19 GEA patients from the cBioPortal database; (**C**) The most common CNVs of 19 GEA patients from the cBioPortal database; (**D**) CNV profiles of 19 GEA patients from the cBioPortal database; (**E**) Comparison analysis of mutation frequency between GEA, HPVI-non GEA, UEA, and SCC. (**F**) Comparison analysis of CNV frequency between GEA, HPVI-non GEA, UEA, and SCC.

Genomic sequencing was recommended to all GEA patients treated at our institution, and due to cost considerations, 17 patients ultimately underwent sequencing. Preliminary analysis in our center showed that genomic changes altered TP53 (59%), PTEN (29%), CDKN2A (35%), and STK11 (35%) (Figure 4A). TP53 mutation (*n* = 10, 59%), PTEN deletion (*n* = 6, 29%), and STK11 mutation (*n* = 5, 29%) might be the most prevalent genomic alterations according to data from our center. Moreover, for more genomic features of GEA, the mutation and CNV data of the online cohort was summarized and compared with SCC and HPVI-non GEA. According to cBioPortal online data, the most common mutation of the GEA cohort might be the TP53 mutation (52.6%), followed by KRAS (26.3%), STK11 (15.8%), and CDKN2A (15.8%) (Figure 4B). The most prevalent CNVs of 19 GEA patients might be KDM6A amplification (10.5%), CDKN2A amplification (10.5%), and CDKN2B amplification (10.5%) (Figure 4C). Online CNV profiles indicated that most GEA patients had a low degree of chromosome-level CNV (Figure 4D). Most deletions were enriched in chromosome 4 (Chr4), Chr9, and Chr18, with the highest gains being in Chr19 and Chr2. Comparison analysis showed that the frequency of TP53 mutation, CDKN2A mutation, KDM6A amplification, and CDKN2A deletion was significantly higher than that of UEA, SCC, and HPVI-non GEA according to the online database (Figure 4E,F).

## 4. Discussion

To the best of our knowledge, this study was the largest single-center study published to date, with detailed information on diagnosis, treatment, oncological outcomes, prognosis analysis, and genomic alterations in GEA. More than 60% of GEA patients showed no specific vaginal bleeding, and approximately 80% showed no specific tumor mass. Nearly 55% of cases were diagnosed during the late stage. Most primary cases received surgery, and most relapsed cases received chemotherapy with or without targeted therapy and immunotherapy. For oncological outcomes, the 5-year survival was 57%, and the recurrence rate was 23% for whole cohort. Survival analysis showed that deep stroma infiltration, ovary metastasis, and ≥3 cm of maximum diameter suggested worse OS and PFS in GEA. For genomic information, TP53 mutation, PTEN deletion, CDKN2A mutation, and STK11 mutation were the most common alterations, which might provide a reference for targeted therapy in GEA patients.

For diagnosis, in this study, the median age at diagnosis is 49 years, which is somewhat younger than previous GEA cohorts [5,19]. More than 21% of GEA patients had no complaints, and nearly 80% of cases presented with no distinct mass through bimanual or rectovaginal examination. Approximately 60% of patients showed no lesions according to TCT. Hence, more than 64% of cases were at stage IIB–IV at diagnosis because the GEA population is very difficult to identify. Previous studies also showed that GEA was usually misdiagnosed as non-cervical diseases (such as ovarian cancer and endometrial lesions) [12,13,14]. In this study, 15 patients presented with primary ovarian cancer or endometrial cancer, arousing suspicion, and 6 patients obtained an accurate diagnosis after performing pathologic examinations three or four times. All this information indicates the significance of clinical inquiry and multicystic features via MRI, as well as the necessity of multifocal or deep pathologic examinations for suspected GEA cases.

For treatment, risk factors might be crucial for regimen design. Consistent with other studies (15–35%), the rate of ovary metastasis was 29% in our study [18,19], which indicated a lower probability of ovarian preservation during surgery, even for younger patients. Moreover, lymph node metastasis was detected in 42% of our cohort through pathologic or imaging examination. Hence, for GEA patients, an evaluation of ovary/lymph node metastasis via PET/CT and careful exploration of abdominal and pelvic lesions in surgery were necessary to improve survival. In particular, due to the potential relationship between ovary metastasis and worse OS/PFS in the whole patients, it is important to consider whether ovarian metastasis reflected advanced disease burden in GEA. High frequency of ovarian metastasis in GEA and the additional value of ovarian metastasis in early-stage indicated that fertility preservation is challenging for GEA with early-stage. Moreover, considering the higher frequency of ovary metastasis compared with UEA and SCC [19], the treatment design for the subpopulation with ovarian metastasis remains to be assessed in future works using more robust comparative or prospective evidence.

For therapy regimen, previous studies indicated a limited response to CT or RT in GEA compared with UEA and SCC, especially for late-stage and local recurrence [19]. In this cohort, only 52% of cases achieved complete remission up to the end of the follow-up period, and recurrence occurred in 23% of patients, with shorter median TTR (9.3 months) than in previous data [7]. Overall, for stage I–II, postoperative therapy was not significantly related to survival outcomes. For stage III–IV, surgery might be related to better overall survival. This potential correlation might indicate that the treatment regimen of GEA at a late stage might be different from SCC; among those, stage III–IV patients mainly chose CCRT-dominated therapy. However, due to the retrospective study design, many baseline parameters potentially affecting treatment outcomes cannot be obtained for comparison analysis. Overall, the treatment choice of GEA still remains to be explored based on prospective studies.

For genomic alteration, preliminary exploratory analysis of genomic data from our center and online database indicated that the alteration of TP53, HER2, and STK11 might be common in GEA, consistent with previous works [26]. Particularly, STK11 mutation and HER2 amplification were reported to correspond to poor survival of GEA patients [35]. However, no significant association was observed between these genomic changes and survival in our GEA cohort, partially due to limited sample size (*n* = 17) and high proportion of stage III–IV (11/17, 64.71%). In future works, the clinical significance and biological implications of these genomic alterations warrant continued exploration. Moreover, genomic information also provides the inspiration for precision therapy for GEA in the future. In previous research, the HER2-targeting inhibitor enriched therapy alterations in advanced/recurrent GEA with HER2 overexpression [27,28]. Previous studies have also shown that some GEA cases carried BRCA1 or BRCA2 mutations, which partially indicated the opportunity of PARP inhibitor for individualized therapy [15,36]. Considering the rarity of GEA and its strong genomic heterogeneity, individualized regimens and clinical trials in HPV-independent cervical cancer might appropriately incorporate findings from genomic profiles, especially for the recurrent or resistant subpopulation. This is especially important for GEA, a more dangerous and rare disease than SCC, with a high frequency of recurrence rate and late-stage diagnosis.

This study does have some limitations. First, its long temporal span means that therapeutic and diagnostic strategies for GEA were not uniform, due to advances in knowledge and variable availability of key tests (like immunohistochemistry) over time. Second, the retrospective design inherently limited data completeness and made it difficult to clarify the significance of certain risk factors. Third, retrospective design may introduce bias in the analysis of treatment regimens. Future prospective studies are warranted to collect comprehensive patient data and draw more definitive conclusions. Moreover, information on targeted therapy and immunotherapy was limited due to the relatively short follow-up period in this study.

Nonetheless, the large sample size, clear therapy regimen, and detailed genomic information in this research should provide inspiration for clinicians with regard to GEA management due to the rarity of GEA and the increasing prevalence of HPV-independent cervical cancer. Clinical trials and retrospective analyses of these regimens are encouraged in future works on HPV-negative cervical adenocarcinoma.

## 5. Conclusions

In conclusion, this study enriched the genomic features, therapy regimens, and survival analysis of GEA. Late-stage diagnosis and a high frequency of recurrence are typical characteristics of GEA. For GEA treatment, it was found that chemotherapy or radiotherapy alone rarely realize complete remission, and surgery was associated with improved overall survival for stage III–IV patients. Survival analysis identified deep infiltration depth (≥2/3), ovary metastasis, and MDOT ≥ 3 cm as indicators of poor OS and PFS. Moreover, genetic heterogeneity in GEA patients may increase regimen alterations, especially for the advanced and recurrent subpopulations. Overall, this single-center study has important implications for GEA management.

## Figures and Tables

**Table 1:** Summary of clinicopathological information of 182 GEA patients.

Age (Year)	49 (Median)	24–81 (Range)
Clinicopathological Parameters	*n*	%
Clinical presentation		
Watery discharge	64/182	35.16%
Vaginal bleeding	72/182	39.56%
Abdominal mass/abdominal distension	7/182	3.85%
Physical examination	39/182	21.43%
Clinical examination		
Cervical hypertrophic or barrel-shaped	79/182	43.41%
Cervical harden	49/182	26.92%
Cervical neoplasm	39/182	21.43%
Cervical smooth	15/182	8.24%
HPV *		
Positive	17/136	12.50%
Negative	119/136	87.50%
TCT *		
Adenocarcinoma	6/135	4.44%
ASC/LSIL/HSIL	25/135	18.52%
AGC	24/135	17.78%
NILM	80/135	59.26%
FIGO stage		
I	59/182	32.42%
II	23/182	12.64%
III	67/182	36.81%
IV	33/182	18.13%
Tumor maximum diameter ^#^		
<3 cm	73/139	52.52%
≥3 cm	67/139	48.20%
Stromal invasion ^#^		
<2/3	54/139	38.85%
≥2/3	85/139	61.15%
Lymphovascular space invasion ^#^		
Present	69/139	49.64%
Absent	70/139	50.36%
Lymph node metastasis		
Present	63/150	42.00%
Absent	87/150	58.00%
Ovary metastasis ^#^		
Present	41/140	29.29%
Absent	99/140	70.71%
IHC *		
MUC6 positive	101/110	91.82%
P53 mutation	44/130	33.85%
P53 wild-type	71/130	54.62%
P53 negative	15/130	11.54%

*: Data for some examination results were not available in the digital record due to incomplete patient recall and the fact that some patients did not perform HPV testing or TCT. ^#^: These data fields were only summarized in patients undergoing surgery with available pathological confirmation. Regarding ovarian metastasis, one patient underwent adnexal biopsy and cervical biopsy and finally pathologically confirmed GEA, without receiving radical hysterectomy. Abbreviations: Thinprep cytologic test, TCT; atypical glandular cells, AGC; atypical squamous cells, ASC; low-grade squamous intraepithelial lesion, LSIL; high-grade squamous intraepithelial lesion, HSIL; negative for intraepithelial lesion or malignancy, NILM.

**Table 2:** Primary treatment and disease status of GEA patients.

Treatment Regimen	*n*	%
Surgery alone	13/182	7.14%
Surgery with postoperative adjuvant treatment	108/182	59.34%
CCRT	31/182	17.03%
SCRT	60/182	32.97%
Chemotherapy	13/182	7.14%
Immunotherapy involved	28/182	15.38%
Targeted-therapy involved	20/182	10.99%
Neoadjuvant therapy followed by surgery with postoperative adjuvant treatment	22/182	12.09%
NACT with hysterectomy with CCRT or SCRT	17/182	9.34%
Neoadjuvant CCRT followed by hysterectomy with chemotherapy	5/182	2.75%
Without surgery	43/182	23.63%
CCRT	25/182	13.74%
SCRT	12/182	6.59%
Chemotherapy	6/182	3.30%
Immunotherapy involved	6/182	3.30%
Targeted-therapy Involved	11/182	6.04%
Relapse	41/182	22.53%
Vagina	8/182	4.40%
Lung	14/182	7.69%
Bowel	14/182	7.69%
Lymph node	5/182	2.75%
Without relapse	141/182	77.47%
Disease Status	182/182	100.00%
NED	95/182	52.20%
AWD	38/182	20.88%
DOD	49/182	26.92%

Abbreviations: NED, no evidence of disease; AWD, alive with disease; DOD, dead of disease.

**Table 3:** Analysis of OS and PFS prognostic factors for GEA.

Characteristic	Comparator	Univariate Analysis (OS)		Multivariate Analysis (OS)		Univariate Analysis (PFS)		Multivariate Analysis (PFS)	
		HR (95% CI)	*p* Value	HR (95% CI)	*p* Value	HR (95% CI)	*p* Value	HR (95% CI)	*p* Value
Age	>50 vs. ≤50	0.99 (0.48–2.01)	0.980	1.26 (0.55–2.91)	0.585	1.13 (0.65–1.96)	0.662	1.44 (0.77–2.71)	0.256
FIGO Stage	IIB–IV vs. I–IIA	5.33 (2.17–13.09)	< 0.001 *	3.81 (1.13–12.80)	0.031 *	3.45 (1.87–6.36)	0.000 *	1.71 (0.72–4.04)	0.224
MDOT	≥3 cm vs. <3 cm	3.56 (1.53–8.27)	0.003 *	3.73 (1.48–9.37)	0.005 *	3.26 (1.79–5.93)	0.000 *	2.59 (1.39–4.83)	0.003 *
LVSI	Positive vs. negative	0.89 (0.44–1.79)	0.745	0.38 (0.16–0.91)	0.030 *	1.56 (0.90–2.71)	0.116 *	1.03 (0.55–1.93)	0.917
Infiltration depth	≥2/3 vs. <2/3	2.30 (1.06–4.98)	0.035 *	2.79 (1.10–7.11)	0.031 *	2.20 (1.23–3.96)	0.008	2.02 (1.09–3.73)	0.026 *
Lymph node metastasis	Positive vs. negative	3.28 (1.61–6.68)	0.001 *	1.30 (0.44–3.80)	0.643	2.80 (1.63–4.81)	0.000 *	1.50 (0.67–3.32)	0.324
Ovary metastasis	Positive vs. negative	6.50 (3.05–13.80)	<0.001 *	3.62 (1.61–8.16)	0.002 *	3.80 (2.19–6.58)	0.000 *	2.61 (1.41–4.81)	0.002 *

*: *p* < 0.05.

## Data Availability

The datasets analyzed during the current study are available on the cBioPortal website (https://www.cbioportal.org/). All data generated or analyzed from PUMC during this study have been uploaded to the GSA database (https://ngdc.cncb.ac.cn/; accession number: PRJCA049944). Raw data are available from the corresponding author on reasonable request.

## Supplementary Materials

The following supporting information can be downloaded at https://www.mdpi.com/article/10.3390/cancers18020320/s1, Figure S1: Flow chart of the GEA retrospective study; Figure S2: Comparison analysis between patients with or without postoperative therapy in stage I–IIA patients; Table S1: Analysis of OS and PFS prognostic factors for different stage.

## Abbreviations

The following abbreviations are used in this manuscript:

GEA	Gastric-type endocervical adenocarcinoma
UEA	Usual-type endocervical adenocarcinoma
HPVI	HPV-independent
HPVA	HPV-associated
PJS	Peutz–Jeghers syndrome
SCC	Squamous cell carcinoma
AGC	Atypical glandular cells
ASC	Atypical squamous cells
LSIL	Low-grade squamous intraepithelial lesion
HSIL	High-grade squamous intraepithelial lesion
NILM	Negative for intraepithelial lesion or malignancy
