# Supplementary material for: Oncological Outcomes and Genomic Features of Gastric-Type Endocervical Adenocarcinoma, the Most Aggressive and Common HPV-Independent Cervical Cancer
Source: Cancers (Basel). 2026 Jan 20;18(2):320. doi: 10.3390/cancers18020320 (PMC12839000; doi:10.3390/cancers18020320)
Supplement: Supplementary file 1 [file cancers-18-00320-s001.zip › Supplementary Method.pdf]

Variant analysis

All nonsynonymous variants covered more than 20x and within the coding and splicing regions were selected and screened with dbSNP, 1000 Genomes Project (1000G), NHLBI Exome Sequencing Project (ESP6500), and Exome Aggregation Consortium (ExAC). Potential
functional consequences of the nonsynonymous variants were evaluated by SIFT, Mutationtaster, and PolyPhen-2, and CADD. The variants known to be pathogenic to the disease in any database of NCBI ClinVar, HGMD
(Human Gene Mutation Database)， and OMIM were picked out for further analysis.

SNV and INDEL calling. (Mutect2)

Sequencing reads were aligned to the reference genome hg19 build (UCSC) using BWA (BWA-0.5.9) with default parameters to generate a
binary sequence alignment map (BAM) file . The aligned BAM file was sorted and merged using Samtools (v1.3.1), and the duplications were marked and removed using Picard tools(v2.6.0). Then, realignment of all insertions and deletions (INDELs) and recalibration of base quality were done with Mutect2() . We set the following criteria for identification of reliable somatic SNVs or INDELs: (1) reads covering the mutated sites should number more than 10, with at least 3 reads harboring the mutations; (2) reads covering the mutated sites in the corresponding normal control should number more than 10, with at most 1 read harboring the mutations; (3) mutations listed in dbSNP 135 were removed unless they were documented by the Catalog of Somatic Mutations in Cancer (COSMIC);

and (4) mutations listed in the National Heart, Lung, and Blood Institute Exome Sequencing Project were excluded.

SNV and INDEL calling(VarScan2)

Sequencing reads were aligned to the reference genome hg19 using BWA (v0.7.10) with default parameters to generate a binary sequence alignment map (BAM) file . The aligned BAM file was sorted and merged using Samtools (v1.3.1), and the duplications were marked and removed using Picard-tools(v2.6.0). Then, somatic SNV and small indels were identified through VarScan2(v2.3.6) . Somatic SNVs were further filtered by VarScan2 internal threshold, and those SNVs that failed the threshold were excluded. Somatic SNVs and indels inferred by VarScan2 software were filtered following the criteria: 1) Read depth ≥ 20 in both tumor and normal samples, 2) Read support of mutant allele in tumor tissue not as a result of sequencing error (Binomial test, $p > 0.01$), 3) Quality score not significantly lower than other alleles (Wilcoxon rank sum test, $p > 0.01$), 4) Mutant allele frequency change between tumor and adjacent normal ≥ 20% and Fisher's Exact Test p-value < 0.01, 5) Mutant allele not significantly enriched in repeatedly aligned reads,6) Mutant allele not significantly enriched within 10 bps of 5′ or 3′ ends of reads (Fisher's exact test, $p > 0.01$) and 7) Mutant allele were observed in both forward and reverse strand of the tumor DNA. The resulting mutation were annotated by annovar. Variants fulfilled the following criteria were first excluded: a). the variant did not affect the amino acid; b). allele frequency > 5% in 1000 Genomes Project, ESP database or gnomAD. c) the variant benign or likely benign were reported by clinvar or intervar.

Determination of potential driver mutations.

To identify potential driver mutations, we evaluated the non-synonymous mutations from the following three aspects: (1) Mutations in significantly mutated genes highlighted by recent large cohort sequencing studies ; (2) Mutations in genes that are documented by the COSMIC database; (3) Mutations in genes that are present in the KEGG (Kyoto Encyclopedia of Genes and Genomes) pathways in cancer. Putative driver mutations were determined if they matched one of the
above requirements.
