# Supplementary material for: Oncological Outcomes and Genomic Features of Gastric-Type Endocervical Adenocarcinoma, the Most Aggressive and Common HPV-Independent Cervical Cancer
Source: Cancers (Basel). 2026 Jan 20;18(2):320. doi: 10.3390/cancers18020320 (PMC12839000; doi:10.3390/cancers18020320)
Supplement: Supplementary file 1 [file cancers-18-00320-s001.zip › Supplementary Table 1.pdf]

Supplementary Table S1. Analysis of OS and PFS prognostic factors for different stage.

	Characteristic	Comparator	Multivariate analysis (OS)		Multivariate analysis (PFS)	
			HR (95% CI)	P value	HR (95% CI)	P value
Stage I-II	Age	＞50 vs. ≤ 50	0.40 (0.07-2.21)	0.292	0.50 (0.17-1.47)	0.209
	Postoperative treatment	With vs. without	0.14 (0.01-1.66)	0.120	2.19 (0.47-10.15)	0.315
	Ovary metastasis	With vs. without	17.39 (1.87-162.19)	0.012*	3.75 (1.19-11.81)	0.024*
Stage III-IV	Age	＞50 vs. ≤ 50	1.29 (0.66-2.52)	0.454	2.00 (1.14-3.52)	0.016*
	Surgery	With vs. without	0.48 (0.25-0.95)	0.036*	0.98 (0.54-1.79)	0.953
Stage I-IV	Age	＞50 vs. ≤ 50	1.22 (0.67-2.21)	0.521	1.42 (0.88-2.28)	0.15
	Surgery	With vs. without	0.55 (0.29-1.04)	0.067	0.87 (0.50-1.52)	0.63
	Stage	IIB-IV vs I-IIA	5.14 (2.09-12.63)	<0.001*	3.66 (1.97-6.79)	<0.001*
