# Supplementary figures and images for: Oncological Outcomes and Genomic Features of Gastric-Type Endocervical Adenocarcinoma, the Most Aggressive and Common HPV-Independent Cervical Cancer
Source: Cancers (Basel). 2026 Jan 20;18(2):320. doi: 10.3390/cancers18020320 (PMC12839000; doi:10.3390/cancers18020320)

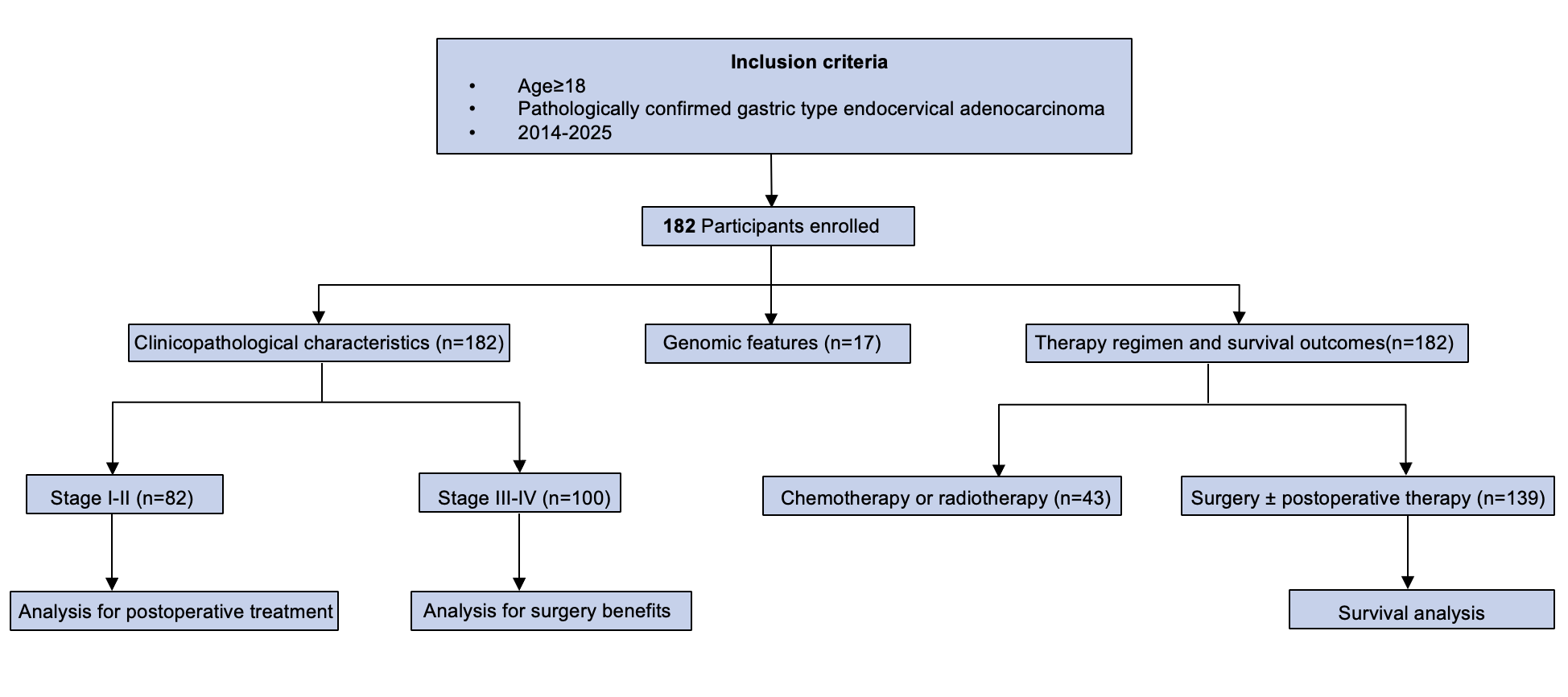

Supplement: Supplementary file 1 [file cancers-18-00320-s001.zip › Supplementary Figure 1.tif]

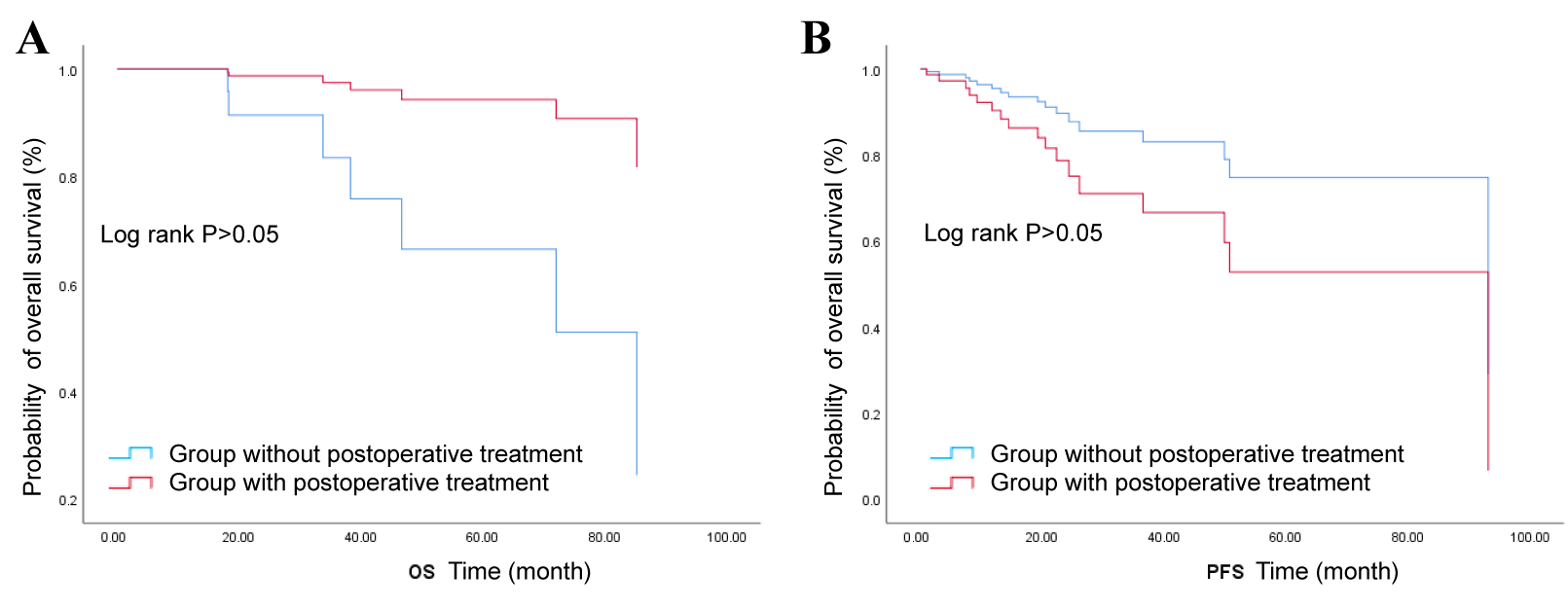

Supplement: Supplementary file 1 [file cancers-18-00320-s001.zip › supplementary figure 2.tif]
